# Toxicity of Buprofezin on the Survival of Embryo and Larvae of African Catfish, *Clarias gariepinus* (Bloch)

**DOI:** 10.1371/journal.pone.0075545

**Published:** 2013-10-03

**Authors:** Kasi Marimuthu, Narmataa Muthu, Rathinam Xavier, Jesu Arockiaraj, M. Aminur Rahman, Sreeramanan Subramaniam

**Affiliations:** 1 Department of Biotechnology, Faculty of Applied Sciences, AIMST University, Bedong, Kedah Darul Aman, Malaysia; 2 Division of Fisheries Biotechnology and Molecular Biology, Department of Biotechnology, Faculty of Science and Humanities, SRM University, Kattankulathur 603 203, Chennai, Tamil Nadu, India; 3 Laboratory of Marine Biotechnology, Institute of Bioscience, Universiti Putra Malaysia, 43400 UPM Serdang, Selangor, Malaysia; 4 School of Biological Sciences, Universiti Sains Malaysia (USM), Penang, Malaysia; Dauphin Island Sea Lab, United States of America

## Abstract

Buprofezin is an insect growth regulator and widely used insecticide in Malaysia. The present study evaluated the toxic effects of buprofezin on the embryo and larvae of African catfish (*Clarias gariepinus*) as a model organism. The embryos and larvae were exposed to 7 different concentrations (0, 0.05, 0.5, 5, 25, 50 and 100 mg/L) of buprofezin. Each concentration was assessed in five replicates. Eggs were artificially fertilized and 200 eggs and larvae were subjected to a static bath treatment for all the concentrations. The mortality of embryos was significantly increased with increasing buprofezin concentrations from 5 to 100 mg/L (*p<* 0.05). However, the mortality was not significantly different (*p<*0.05) among the following concentrations: 0 (control), 0.05, 0.5 and 5 mg/L. Data obtained from the buprofezin acute toxicity tests were evaluated using probit analysis. The 24 h LC_50_ value (with 95% confidence limits) of buprofezin for embryos was estimated to be 6.725 (3.167-15.017) mg/L. The hatching of fish embryos was recorded as 68.8, 68.9, 66.9, 66.4, 26.9, 25.1 and 0.12% in response to 7 different concentrations of buprofezin, respectively. The mortality rate of larvae significantly (*p<*0.05) increased with increasing buprofezin concentrations exposed to 24-48 h. The 24 and 48 h LC_50_ values (with 95% confidence limits) of buprofezin for the larvae was estimated to be 5.702 (3.198-8.898) and 4.642 (3.264-6.287) mg/L respectively. There were no significant differences (*p>*0.05) in the LC_50_ values obtained at 24 and 48 h exposure times. Malformations were observed when the embryos and larvae exposed to more than 5 mg/L. The results emerged from the study suggest that even the low concentration (5 mg/L) of buprofezin in the aquatic environment may have adverse effect on the early embryonic and larval development of African catfish.

## Introduction

Globally pesticides play a major role in sustaining the agricultural production by protecting all agricultural crops from pest attack, weeds and vector-borne diseases [1,2]. Rice is the most important cereal crop in the economy of Malaysia. This sector uses huge amount pesticides for controlling pests and plant pathogens. Various categories of pesticides are frequently used to control insect pests. Although the pesticides are often very effective, many of them represent a potential hazard and their use worldwide give rise to concern on health and environmental effects. Some of the pesticides persist for a very long time in the environment and their impacts on the local fauna are largely unknown. Among the pesticides, buprofezin is commonly used in paddy fields for controlling insect pests. These chemicals can find their way into the water reservoirs, streams and rivers, thus producing an adverse impact on the aquatic biota, including fish and other aquatic organisms [3-8].

Buprofezin, (2-tert-butylimino-5-phenyl-3-propan-2-yl-1,3,5-thiadiazinan-4-one) is a thiadiazine insect regulator, molting inhibitor, and it acts specifically on immature developmental stages of homopteran pests by inhibiting the incorporation of N-acetyl-[D-H3] glucosamine into chitin and interfering with cuticle formation resulting in nymphal mortality during molting [9,10]. It also exhibits larvicidal activity against the brown rice planthopper, *Nilaparvata lugens* and the greenhouse whitefly, *Trialeurodes vaporariorum* [11,12]. Buprofezin exhibits low acute toxicity by oral, dermal or inhalation routes in rat. The oral LD_50_ in rats is reported to be 3847 and 2278 mg/kg body weight (bw) in males and females respectively [13]. Nevertheless, the acute toxicity of buprofezin in aquatic organisms is lacking [14,15].

The toxicological effects of pesticide pollution on non target aquatic organisms in the environment can be examined by detecting changes in organisms at the developmental, physiological, biochemical or molecular levels, which can provide biomarker tools in monitoring environment quality [16,17]. The developing fish embryo or larvae is generally considered to be the most sensitive stage in the life cycle of a teleost fish being particularly sensitive to low level of environmental pollutants [18]. These biomarkers can measure the interaction between environmental xenobiotics and biological effects. Inhibition and induction of these biomarkers are a good approach to measure potential impacts of pollutants on aquatic organisms [19]. Most of the catfish farms in Malaysia are located in and around the agriculture fields or their source of water is continuously in contact with the paddy ecosystem in which lot of pesticides, weedicides and insecticides are routinely used. African catfish is a high fecund fish species, can be easily bred and maintained in laboratory. The candidate fish is very popular and farmed fish species in Malaysia and commonly found in paddy fields. To the best of our knowledge there is no earlier studies so far have been conducted to understand the toxic potential of buprofezin in fishes commonly found in paddy ecosystem. Keeping in view of these aspects, a study was conducted to evaluate the potential effects of buprofezin on the embryo and larvae of African catfish, *Clarias gariepinus*. The data reported in this study would be useful for the management of paddy ecosystem with respect to the regulation of buprofezin usage in rice fields.

## Materials and Methods

### Brood fish and buprofezin

This study was carried out at the aquaculture research laboratory, AIMST University, Kedah, Malaysia. The tap water was stored in a circular storage tanks for at least 48 h to remove chlorine before use for the experimental purposes. Sexually matured male and ripe female African catfish weighing 2 to 3 kg, with a length of 40-50 cm were obtained from a local fish supplier, Sungai Petani, Kedah Darulaman, Malaysia. The brood fish were stocked in 500 L circular cement tanks, at 25±1 °C under natural light conditions. The buprofezin was purchased from a local pesticide supplier, Sungai Petani, Kedah Darulaman, Malaysia. The experiment was approved by the AIMST University Human and Animal Ethics committee (AUHAEC 31/FAS/2011), Malaysia.

### Hormone administration, collection of gametes, fertilization and incubation of eggs

For this experiment, the two male and two female fish were selected based on the external morphological features as described by Marimuthu et al. [20]. Matured male fish was identified by a slightly pointed genital papilla, and females by a swollen abdomen and a reddish swollen vent. In addition, the maturity of the ripe female was confirmed by a slight pressing of the ventral side of the fish for oozing of eggs. Both the female and male fish were artificially induced by intra-muscular injection with 0.4 mL of Ovaprim/kg bw. Hormone injected male and female fish were then released separately into circular cement tanks (500 L) containing dechlorinated tap water. After 12 h of hormone administration, eggs were stripped into circular plastic trays and the testes were removed from the male fish and sperm was pressed into a dry sterile petri dish. Stripped eggs were then fertilized with the diluted sperm suspension. After 2 min of gentle stirring, the fertilized eggs were washed several times with fresh water to remove excess milt. The fertilized eggs were immediately placed in experimental units for embryo toxicity assay. A portion of fertilized eggs were released into the glass aquarium to obtain hatchlings for the larval bioassay studies.

### Experimental setup

The stock solution was prepared by dissolving a weighed amount of buprofezin in distilled water. Seven different concentrations (0, 0.05, 0.5, 5.0, 25, 50 and 100 mg/L) for embryonic and larval bioassay were obtained by the addition of buprofezin stock solution. The control group was exposed to tap water. To evaluate the embryonic toxicity, 200 fertilized eggs were randomly selected and exposed to the respective concentrations. Each experimental treatment and control was executed in five replicates. The experiments were conducted using one liter capacity plastic containers. Treatments were allotted at random in the experimental units. Water quality parameters of the reservoir in the experimental media were determined according to APHA [21]. The mean values for experimental water qualities were as follows: temperature 25.0 ± 1.0 °C, pH 7.5 ± 0.2, dissolved oxygen 7.1 ± 0.1mg/L, total alkalinity (as CaCO_3_) 29.0 ± 1.0 mg/L, hardness 35.0 ± 5.2 mg/L, nitrite 0.01 mg/L, nitrate 2.7 ± 0.1 mg/L, chloride 6 ± 1 mg/L and ammonia 0.01 ± 0.001 mg/L. The hatching time and number of dead embryos were recorded for both the control and experimental groups. To study the larval toxicity, 200 hatchlings were placed into each aquarium and each test concentrations and control groups were executed with five replicates. Malformations were described and documented among the embryos and larvae from both the control and treated groups. After 24 and 48 h exposure periods to different concentrations of buprofezin, each container was examined and the numbers of dead larvae were counted. Mortality was defined as enlarged and white opaque larvae that were non-motile and not responding to agitation with a plastic rod. Survival rate of hatchlings was calculated at 24 h and 48 h of exposure in the tested concentrations and control group.

### Statistical analyses

The data on hatching and survival rate presented in this study is the average of five replicates ± standard deviation (SD). The LC_50_ and its associated 95% confidence intervals (95% CI) were calculated by probit analysis using SPSS (Version 13). To evaluate the different concentrations of buprofezin toxic influence in embryo and larvae, a one-way analysis of variance (ANOVA) and mean comparisons were performed by Duncan’s multiple comparison tests using SPSS (Version 13) at the 5% significant level.

## Results

The acute toxicity of buprofezin on the survival of African catfish embryo is presented in Table 1. In the embryonic toxicity assay, the mortality of embryos was significantly (*p <* 0.05) increased with increasing buprofezin concentrations from 5 to 100 mg/L, while these values did not significantly differ among the control, 0.05, 0.5 and 5 mg/L treatment groups. The incubation periods of fertilized eggs were determined as 24 h in the control groups and 25-40 h in the experimental groups. The increased incubation period was noticed in all the experimental groups except at lower concentration of buprofezin (0.05 and 0.5). The 24 h LC_50_ value (95% confidence limits) of buprofezin for embryos was found to be 6.725 (3.167-15.017) mg/L. The hatching success of embryos with a an increasing concentrations buprofezin as follows 0.0, 0.05, 0.5, 5, 25, 50 and 100 mg/L lead to a hatching rate of 68.8, 68.9, 66.9, 66.4, 26.9, 25.1 and 0.12%, respectively (*p <*0.05).

**Table 1:** Acute toxicity of buprofezin on survival of embryo and larvae of African catfish, *Clarias gariepinus*.

Concentrations (mg/L)	Incubation period (hrs)	Hatching rate (%)	Survival rate of hatchlings at 24 hrs	Survival rate of hatchlings at 48 hrs
Control	25.00	68.8±2.1	100	100
0.05	25.00	68.9±4.1	100	100
0.5	25.30	66.9±9.5	99.3 ±1.1	97.9 ±1.6
5	26.00	66.4±8.1	63.8±23.4	63.8±23.4
25	28.00	26.9±3.6	ND	ND
50	32.00	25.1±6.9	ND	ND
100	40.00	0.12±0.2	ND	ND
P value	*<*0.05	*<*0.05	*<*0.05	*<*0.05
LC_50_ value with 95%		6.725	5.702	4.642
confidence limits		(3.167-15.017)	(3.198-8.898)	(3.264-6.287)

ND - no data (100% mortality)

In the larval toxicity assay, the rate of larval survival at different concentrations of buprofezin was examined in relation to the duration (24 and 48 h) of exposure (Table 1). The survival rate was significantly (*p<*0.05) decreased with increasing concentrations from 5 to 100 mg/L exposed for 24 and 48 h while these values did not significantly differ among the control, 0.05 and 0.5 mg/L treatment groups (*p >* 0.05). There were no significant differences in mortality rate of larvae between the exposure times 24 and 48 h in each concentration (*p >* 0.05). The higher concentration of 25 mg/L showed the highest larval mortality at 24 h of exposure. The 24 and 48 h LC_50_ values (with 95% confidence limits) of buprofezin for larvae were estimated to be 5.702 (3.198-8.898) and 4.642 (3.264-6.287) mg/L respectively.

**Figure 1:**
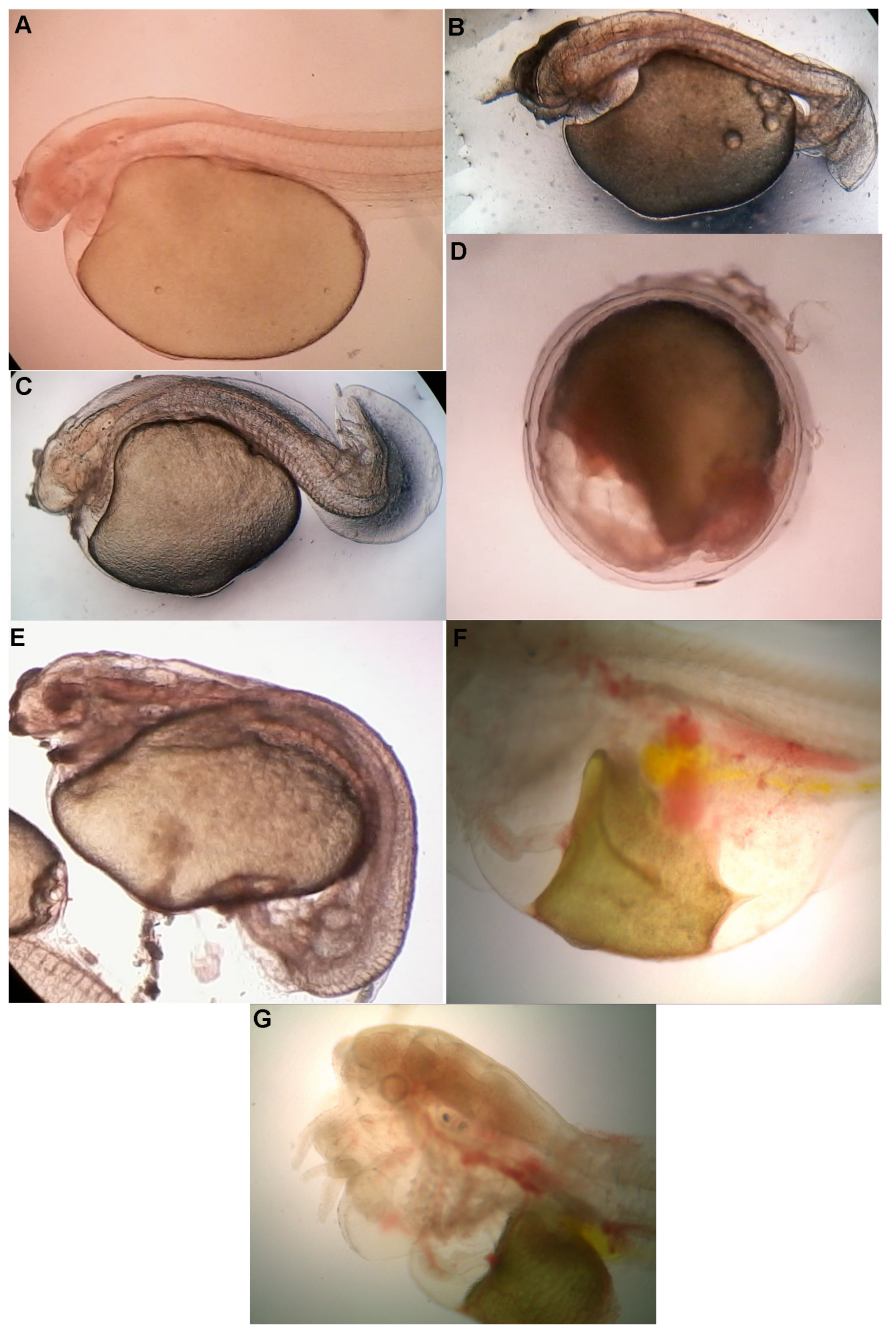
Malformations (irregular head shape, lordosis, yolk sac edema, body arcuation, tissue ulceration, pericardial edema and pericardial hemorrhage) induced by buprofezin. A. 12 hrs old hatchling from control treatment; B. Embryo with irregular head shape and lordosis; C. Notochordal abnormality (body curvature) lordosis; D. Unhatched embryos exposed with 5 mg/L; E. Yolk sac edema and body arcuation in the embryos exposed with 5 mg/L; F. Hatchlings with pericardial edema exposed with 5 mg/L and G.Pericardial hemorrhage exposed with 5 mg/L.

In this study, malformation induced by buprofezin in the embryos and larvae was recorded in the treatment groups. Abnormality was not observed in embryos or larvae exposed to buprofezin at a concentration of *<*0.5mg/L. However, at a concentration of *>*5 mg/L, the embryos and larvae exhibited malformations, characterized by irregular head shape, lordosis, yolk sac edema, body arcuation, tissue ulceration, pericardial edema and pericardial hemorrhage (Figure 1 A-G). Some of the affected embryos were unable to hatch and eventually died (Figure 1D) in the treatment groups.

## Discussion

It has been noticed that increasing buprofezin concentrations had significant effects on hatching success and survival of African catfish embryos and larvae. For example, 68.9% of eggs were hatched when they were exposed to 0.05 mg/L buprofezin, whereas 25.1% hatching rate was obtained in 25 mg/L, and only 0.12% in 100 mg/L buprofezin concentration respectively. The 24 h LC_50_ value of buprofezin for catfish embryos and larvae were found to be 6.725 and 5.702 mg/L respectively. These observations indicate that, buprofezin is highly toxic to embryo and larvae of fishes. The EFSA (European Food Safety Authority) document reported that buprofezin has acute toxicity to fish (rainbow trout and carp) in laboratory tests to be in the average LC_50_ values of 1.4-2.7 mg/L. The 48 h EC_50_ values of buprofezin for zooplankton *Daphnia magna* was 0.44 ± 0.06 mg/L; which is classified as very toxic (≤1mg/L) according to the European Chemicals Bureau and OECD guidelines (European Commission [22] OECD [23]. This result agrees well with the EFSA data, indicating that buprofezin is very toxic to aquatic organisms [16].

In this study, the larvae exposed to increasing buprofezin concentrations significantly increased the mortality rate of larvae. The 100% mortality of larvae was noticed in the 25 mg/L buprofezin concentration. However, no significant difference (*p>*0.05) was noticed in the mortality rates of larvae exposed to the durations of 24 and 48 h (*p>*0.05). In the present finding, the 24 h and 48 h LC_50_ values of buprofezin for African catfish, larvae was found as 5.702, and 4.642 mg/L and here we report buprofezin to be highly toxic to fish larvae. The sensitivity differs from toxicants as well as the developmental stages of fish and some toxicants show higher sensitivity in embryos whereas others are more toxic to larvae [24,25]. Marty et al. found early life stage of *Oryzias latipes* to be the most sensitive phase to toxic effects [26]. Aydin and Koprucu reported that common carp eggs is more sensitive than larvae and also found that the 48 h LC_50_ value of diazinon for common carp embryo was 0.999 mg/L, and 24 and 48 h LC_50_ values of larvae was recorded to be 3.688 and 2.903 mg/L, respectively [27]. Takimoto et al. also reported that timing of exposure of *O. latipes* embryos to sub lethal concentrations of the organophosphate fenitrothion resulted in significantly different degrees of mortality and hatching success [28]. The present results strongly support the earlier findings by Marty et al., Takimoto et al. and Aydın and Koprucu [26-28].

Developmental abnormalities, such as irregular head shape, lordosis, yolk sac edema, body arcuation, tissue ulceration, pericardial edema and pericardial hemorrhage were also found in the group exposed to buprofezin. To our knowledge, the present study is one of the first to evaluate developmental toxicity in fish caused by exposure to buprofezin in aquatic environments. These studies demonstrate that organisms in the early stages of embryonic development are usually more sensitive to toxicological effects. Thus, examining organisms at these stages can help evaluate the sub lethal effect of pesticides and pollutants distinguish the nature of the toxicological effect. Hatching retardation of fish embryos might be due to disturbance of the hatching enzyme and hypoxia induced by buprofezin. During the normal hatching process of fish embryos, the chorion is digested by the hatching enzyme, which is a proteolytic enzyme secreted from hatching gland cells of the embryo. The physiological processes involved as well as mechanism underlying neural control in hatching of fish embryos is remain unclear. However, exposure of teleost embryos to neurotransmitter agonists and antagonists has suggested such a role. Schoots et al. reported that dopaminergic agonists increase the time of hatching while antagonists cause a decrease, whereas Di Michele and Taylor [29,30] reported that epinephrine decreased average time to hatch. In addition, Di Michele and Taylor (1981) demonstrated that atropine, a muscarinic receptor antagonist, inhibited hatching in *Fundulus heteroclitus* [31]. More work is therefore needed to understand the normal biology of the hatching process and how buprofezin interferes with the development of the hatching gland. It has been reported that the high toxicity of pesticides and insecticides in fish is greatly influenced by the combination of three factors such as central nervous system, hydrolytic detoxification, and the route of exposure (direct absorption via the gills into the bloodstream) [32].

Generally insect growth regulators are considered to be much safer to the non-target animals than the other pesticides used in the agriculture sector [33]. This study elucidated the toxicity of buprofezin using African catfish embryos and larvae as a model organism in the aquatic animals and it was found that this type of insecticide has an adverse effect on the hatching success of eggs and survival of fish larvae. At a lower concentration of 25 mg/L it was found that there is a significantly higher mortality of embryo and even 5 mg/L buprofezin substantially reduced the survival of larvae. The results obtained from the present study also indicate that low levels of buprofezin (5 mg/L) in the aquatic environment may have a significant effect on embryo and larvae of African catfish. Attention must be taken pertaining to the adverse effects that buprofezin as well as other similar insecticides might have on non-target aquatic species. Therefore, it could be concluded that buprofezin contamination is dangerous to the aquatic ecosystems, and the issue should be taken into consideration when this insecticide is used in agriculture or in the control of insect populations. In addition, the potential risk from buprofezin metabolites should be investigated to get a more complete picture in terms of toxicity.
